# "The Hospital" Nursing Mirror

**Published:** 1901-03-16

**Authors:** 


"The Hospital" Nursing Mirror.

Being the Nursing Section of "The Hospital."

[Contributions for this Section of "The Hospital" should be addressed to the Editor, "The Hospital" Nursing Mirror, 28 & 29 Southampton Street Strand, London, W.C.]

### Notes on News from the Nursing World.

### THE ROYAL NATIONAL PENSION FUND FOR NURSES.

Members of the Royal National Pension Fund for Nurses in all parts of the world will rejoice that the King and Queen have graciously consented to continue to act as their patron and president respectively. Their Majesties have of necessity been compelled to relinquish some of the positions which they held as Prince and Princess of Wales, and it shows the great personal interest they take in the welfare of nurses that they have resolved to maintain their connection with the Pension Fund—a connection which, as we were able to show on a recent occasion, has been of the utmost practical benefit to the organisation and has largely helped to bring about its remarkable success. The kindness of the Queen is realised and appreciated by none of her subjects more than by the nurses of the Pension Fund, who have personally participated in it at her receptions at Marlborough House.

### THE WAR NURSES.

From Pretoria it is announced that Nursing Sister Ellen O'Neill was dangerously ill on March 7th. The nature of the disease is not stated. Nursing Sister H. Henderson who was reported dangerously ill at Middelburg, Transvaal, on March 5th, was out of danger on March 8th. Nursing Sister A. Nixon, K. N. D. Despard, A. S. Siddons, and L. Lees are coming home in the *Avoca*, which left for England on March 5th, and Nursing Sisters A. Price, H. Galloway, and A. B. Price in the *City of Vienna*, which left on March 3rd. The Secretary of State for War informs us that the following eleven sisters of the Army Nursing Service Reserve proceeded to South Africa in the s.s. *Briton* on the 9th instant:—Misses M. Calverley, A. Campbell, S. Dauney, A. M. Harrison, A. Lawrence, H. H. Mason, C. E. Mernagh, E. T. Powdrell, A. B. Smith, A. M. A. Turner, and A. A. Watson.

### VRYBURG HOSPITAL.

Perhaps no town in South Africa has been more affected by the war than Vryburg. It is in Bechuanaland and 120 miles from Kimberley. The climate is pleasant, though the change of temperature between morning and evening is very trying to the new comer. The hospital, says a correspondent, is a good building containing about 30 beds. The staff consists of a matron, two staff nurses and a probationer. The nurses have a nice sitting-room and separate bedrooms. The matron receives a salary of £130 per annum and the senior staff nurse £97.

### POOR LAW NURSING IN IRELAND.

The opinion of The Macdermott, K.C., has been considered by the Armagh Board of Guardians, who consulted him as to the legality of the recent sealed order issued by the Irish Local Government Board in reference to the nursing regulations, and it is that the latter do not possess the power which they claim to exercise. He therefore advises that an appeal should be made to the Lord Lieutenant, and that in the meantime the order should not be acted upon. He also suggests that other Boards of Guardians should act in concert with that of Armagh. If the suggestion finds favour, we shall have the Poor Law Guardians of Ireland in open rebellion against the Local Government Board. The Armagh Board at their last meeting decided to do their best to bring this about by circularising the other boards, and organising an agitation against the new order. We hope that the Local Government Board are fully prepared to meet the agitation, and to insist upon the order being obeyed. It is inconceivable that they have issued it without the sanction of even higher legal authorities than The Macdermott.

### HOTEL NURSING AND THE "LADY NURSE."

Extremes meet, and an organ of hotel-keepers is as unfair in its comments on nurses as "A Matron of Experience" is unjust in her estimate of the attitude of the clergy and of men of the world towards her vocation. The *Hotel World* says:—

> Apropos of hotel nursing and The Hospital, all the fuss made about nurses and their organisations, &c., is mostly bosh, and certainly injurious. The old monthly has given way to the professional nurse without, we fear, very much improvement. Where the one drank and slept, the other is an epicure and domineers. The worst of all is, however, the lady nurse, who has nothing sisterly about her, except her uniform, and that chiefly with coquettish intentions. The way weak patients are tyrannised over by the modern nurse makes everyone glad to see her leave the house, whilst her exactions of food, *primeurs*, and pretensions of special *service* make her a ruinous terror to all except the very wealthy.

To some extent this retort discourteous has been provoked by the demand of nurses to dine at *table d'hôte* in uniform, and their objection to take their meals with the lady who acts as housekeeper in a large hotel. But while we regret the folly of a few nurses who have brought obloquy upon their vocation, we cannot too severely condemn the caricature of the "lady nurse" which is drawn by our contemporary. It would be just as reasonable to take a fusty, ill-managed, uncomfortable inn as typical, and pronounce all hotels unworthy of support as it is to condemn the modern nurse because she is not always considerate, unselfish, and sensible. We also dissent from the conclusions arrived at by a correspondent who writes to us as "A Mere Outsider" to ask for trained women of a lower class, "who can make themselves generally useful," as nurses. The expression "lady nurse" is unfortunate, for all nurses should be ladies, either by intuition or by education, and no woman who has not the instincts of a lady is fit to be a nurse. But the "lower class," who would compete with lady's maids and housemaids, or general servants, is not at all the class we desire to see engaged in nursing. It is a class, moreover, which has been tried and found wanting. There are plenty of trained nurses, who are also ladies, with no objection to serve in the humblest of abodes; and though we know that the problem of providing nurses for the poorer middle classes is not yet satisfactorily solved, its solution does not lie in the training and organisation of a second grade of nurses.

### AFTER THREE YEARS.

At last the Plymouth Corporation of the Poor have come to their senses. "For three years," said the Governor at a meeting last week, "I have been chairman of the Hospital Committee, and requests from the doctor, the nursing staff, and the committee have been consistently placed before the Board for additional nurses because the sick wards were understaffed, and the requests had been refused." Possibly the reason why at length the Board have given way and sanctioned the appointment of two additional nurses, is that recently two patients practically died in the workhouse infirmary during the time the night nurse left them, and before she was able to get back again, her rounds being so great a distance and her duties so many. We say practically died, because the master of the workhouse said that though one patient died of heart disease before the nurse could see to him, the other was not dead, "but so bad that nothing could be done for her." In the face of these facts, and, also, perhaps, because the new superintendent very properly signalised her accession to office by declaring that it was impossible for the patients to receive proper treatment without more nurses, the prolonged hostility of the Guardians broke down. But the position is by no means satisfactory even now. The Governor describes the workhouse premises as "regular scattered homes," and confirming a statement in "*The Hospital*" *Nursing Mirror* three weeks ago, says that "the nurses, to get from one sick ward to the other, have to walk across open yards, and patients in certain wards have also to cross the yards at times." If it is impracticable to erect a new building with proper provision for patients and nurses, the open yards should at any rate be covered in sufficiently to afford protection against bad weather.

### NURSING IN AMERICA.

The following is an extract from the letter of an English nurse working in America, where training and experience are exceptionally good:—"It is a struggle to get cases out here now unless one trains in this country; I have had a weary time in Chicago, and unless I do better here I shall return home where I am known. Light cases are unheard of here; much more work and longer hours are expected of a private nurse than with us; from 5 a.m. to 10 p.m. was my fate with my last engagement, before I fell ill, besides broken rest with two little patients. I think the difficulty there is in a strange country to get work, unless trained out here, should be known more in England. Even then the competition is very keen, and 'outsiders' are not welcome. Nurses at home little know the difference of the work in the two countries."

### PROGRESS AT PORTSMOUTH.

It is pleasing to learn that the managers of the Portsmouth Association for Nursing the Sick Poor expect to be able to open the new Nurses' Home in June. The increase in the work of the nurses in 1900 was very considerable, and one of the most signal proofs of the value of it is that more than 200 cases of typhoid fever were nursed. Fortunately, the public are not insensible to the claims of the society to their support, and the accounts for the year show a balance in hand of nearly £100. We lately mentioned that the privilege granted for years to the nurses of riding on the trams free of charge, or at reduced rates was to be cancelled. Mr. Alderman Aldrich says that this means an addition of £40 a year to the cost of carrying on operations. The Corporation will doubtless compensate for the withdrawal of the concession by giving a substantial annual donation to the Association.

### THE COSTUME OF NUNS.

With sneers at nuns as hospital nurses we can have no sort of sympathy. In their individual capacity nuns have often proved most capable and most devoted nurses. To-day there are a great many hospitals in foreign countries, and some in Ireland, as well as in our own colonies, which still depend entirely upon their services. But the protest which has been made in Paris against the costume worn by nuns in nursing is reasonable enough. The large head-dress and hanging sleeves of the sisters of St. Vincent de Paul are in striking contrast with the neat close-fitting print frock, the washing cap and apron of the English hospital nurse; and there can be no dispute as to the advantages of the latter in the interests of hygiene. So far as the charges of proselytism are concerned on the other side of the Channel, it must be remembered that it is generally a question of the Roman Catholic religion or of none; but, of course, anything in the shape of proselytism by nurses is open to objection.

### A NURSE WANTED AT NORTHALLERTON.

At at inquest on the body of James Dalton, a stonemason, who was found dead in his bed from progressive paralysis at Northallerton, the coroner said that "if the man had had a nurse it was probable that there would have been no work for the jury to do." In other words, the unfortunate man died because there is no nurse provided by the parish to attend such cases. Dr. Yeoman, who saw the deceased the night before he died, and sent him medicine, stated that he fully expected to see him alive the next day. No one seems to have been personally to blame for the falsification of his expectation, but it is the duty of the authorities at Northallerton to see that such an incident is not repeated from a similar cause. The town is surely able to support a parish nurse.

### AMERICAN NURSES ON STRIKE.

An incident is reported from Chicago which merits attention. A nurse in a private non-contagious hospital was assigned to a patient, and the nurse, on learning that the case was scarlet fever, refused to remain on duty. For this act of disobedience she was discharged; and the other nurses in the hospital at once "went on strike," alleging that their colleague had been unjustly treated, inasmuch as contagious cases were not usually received in the hospital. Supposing it was well understood that cases of infectious diseases were not received at the hospital, and that the proprietor took in a case of scarlet fever for the sake of the fees, his action would be a gross breach of faith in respect to the other patients. In such a case the refusal of the nurses to remain in the hospital might be laudable, instead of unjustifiable. Of course in ordinary circumstances nothing could excuse a strike of hospital nurses. Any nurse who agrees to join in a strike proves her utter unfitness for her work, which, though it is entitled to adequate remuneration, is not a mere matter of sale and barter. Women who can wilfully neglect the claims of the suffering in order to enforce their own rights are too selfish, if not too cruel, to be trusted with the care of the sick.

### THE NURSES' UNION "AT HOME."

On Thursday last Miss Dashwood, honorary secretary to the Nurses' Union, gave her annual "at home" at her residence in Bryanston Square. The guests were asked to come in indoor uniform, and a dressing-room was set apart for their use. Maids were busy labelling each cloak and bonnet, so that there was no possibility of mistakes. It was quite a sight to see the spacious drawing-room filled with nurses in costumes varied as the rainbow; caps of all shapes and sizes; the white aprons spotless and the collars and cuffs, a connecting link between all. Dr. Pierce Gould, from the Middlesex Hospital, gave a short address, and spoke in the highest terms of the benefit of belonging to such a union. He said that a Christian nurse ought to show by her work what she was, and urged all to be thorough in little things. Nurse Lane followed with an interesting account of her work as a medical missionary for seven years in China.

### ACTION FOR BREACH OF PROMISE BY A NURSE.

At the Breconshire Assizes last week Miss Rhoda Elizabeth Loseby, a district nurse at Bwlch, sued a Calvinistic Methodist minister named Davies for breach of promise of marriage. The evidence indicates that the minister was mercenary, for it transpired that soon after he gave her an engagement ring he asked her "how much money she had." Her reply was that she had not much, and, according to her statement, Mr. Davies "seemed disappointed," and said, "You must not tell anybody of our engagement, as the members of the church would not like it." Subsequently, before starting for a tour in Palestine, he tried to get back the engagement ring he had given her and the letters he had written, but failed. In January 1900 he returned from Palestine, and Miss Loseby confessed that she was so incensed at his conduct that she called him a scamp. His attentions to her then ceased, and an action was entered against him. The plaintiff was awarded £100 damages.

### THE LATE MATRON OF THE SUFFOLK CONVALESCENT HOME.

In consequence of the resignation of Mrs. Pryke, the Committee of the Suffolk Convalescent Home, Felixstowe, require a new matron. Mrs. Pryke's retirement, we regret to say, is due to ill-health after 21 years' arduous work. Trained at University College Hospital, and enjoying subsequently the advantage of considerable experience in the Colonies, she succeeded Miss Reeve in 1879, and at once began to show the energy which has been a characteristic of her administration. Her earliest innovation was to institute sales of work on the beach at Felixstowe, which have since been an annual feature of the season, obtaining a clear profit of £45 on the first occasion. In 1884 the enlargement of the Home, consisting of a handsome wing for men, was finished, and in August 1889 the corresponding wing for women was completed. At the sale of work in August £100 was realised, and though in this and every effort Mrs. Pryke had valuable assistance given to her, which she always gratefully acknowledged, the institution is greatly indebted to her for its now exceedingly prosperous position. Last year 475 patients were admitted, or readmitted, to the Home, and since its opening in 1868 no less than 9,168 persons of all ages have been afforded a temporary asylum within its walls, a large percentage of whom were discharged "quite well."

### QUEEN VICTORIA'S NURSES AT WELSHPOOL.

At the annual meeting of the Victoria Nursing Institute Dr. Hawksworth not only advocated the employment of an additional nurse, but said he hoped "that as each annual meeting came round they would be able to add another nurse." Dealing with the question of difficulties he made light of them, and declared that "he was not one who believed in the policy of cutting the coat according to the cloth. If they wanted the coat most of them could find the money to get it with." Such cheery optimism is refreshing, and in this case it had a solid justification. Lady Powis, who presided at the meeting, had intimated that in order to secure a second nurse she would be willing to double her subscription, and Dr. Hawksworth expressed himself confident that if money were required it could be left to the ladies to find. The manner in which the ladies present at the meeting received his remarks was distinctly encouraging, and there is every reason to anticipate that an early outcome of the Nursing Institute at Welshpool will be the erection of a cottage hospital as an extension.

### CAWNPORE HOSPITAL.

It is interesting to hear from Cawnpore that the work in connection with the hospital of the Women's Medical Association is progressing and is very full of promise." Miss Edmonds—the doctor in charge—was ill with enteric in the spring, but is now back in the hospital. She reports that the out-patient attendance increases, and that a beginning is being made with in-patients, not only with native Christians, but also with non-Christians. The in-patients must sometimes be a little trying, as they will not always stay to be cured. They either become bored and depart, or perhaps their husbands insist on taking them away. This is very disappointing for the hospital staff, but doubtless as the natives begin to understand the advantages of medical treatment they will be more willing, to allow themselves to be properly "doctored" and nursed.

### THE MEATH HOME OF COMFORT.

The report of the working committee of the Meath Home of Comfort for 1900 gives a *résumé* of the development of the work done. With regard to the nursing staff in October 1891, when it began, there were only the lady superintendent and one nurse. The single nurse is now the assistant matron, and there is a staff of twelve nurses with 75 patients to tend, besides the holiday cases received during the summer. The "cottage by the sea" has become a valuable adjunct to the Home for Epileptic Women and Girls at Godalming, and since last summer up to December parties of six, in charge of one of the nurses, enjoyed the advantage of a month's stay at Hayling. It will be a more important adjunct still before long, for Lady Meath has purchased a site on the island and a permanent seaside home, accommodating twelve patients, will be available during the present year. The medical officer bears testimony to the skill and efficient training of the nursing staff, which in August was specially manifested in the case of a most successful operation of a large ovarian tumour, the patient enjoying uninterrupted recovery.

### SHORT ITEMS.

On Tuesday at the Court at St. James's Palace, one of the ceremonies was the presentation by the King of the badge of the Royal Red Cross to Deaconess Jessie Ransome for services rendered at the International Hospital, Peking, during the siege.—On Tuesday afternoon a meeting was held at the Egyptian Hall of the Mansion House to inaugurate the Jubilee year of work of the Zenana Bible and Medical Mission in India. Some interesting addresses were delivered, and Miss Cornall described the medical and missionary work which the mission is carrying on.

### Lectures to Nurses on Medicine and Medical Nursing.

By NORMAN DALTON, M.D. London, F.R.C.P., Physician to King's College Hospital.

(*Continued from page* 284.)

### LECTURE VI. DROPSY.

THE blood consists of enormous quantities of minute solid bodies, called blood corpuscles, suspended in a fluid called the liquor sanguinis. Blood serum is a fluid derived from the liquor sanguinis, and it is colourless because it contains no red blood corpuscles. "Dropsy" is a condition in which the blood serum escapes through the walls of the capillaries into the loose tissue which surrounds those vessels or into any of the serous cavities of the body, such as the pleura, the pericardium, &c. The most conspicuous form of dropsy is the one which occurs in the loose tissue under the skin. This form is called œdema. If the dropsy affects nearly the whole of the skin and the serous cavities as well the condition is called general anasarca. If it occurs in the lungs it is called œdema; in the peritoneal cavity it is called "ascites"; in the pleura, hydrothorax; in the pericardium, hydro-pericardium; and in the tunica vaginalis, hydrocele. Dropsy in the ventricles of the brain forms one variety of hydrocephalus, but the condition here is more complicated.

Dropsy under the skin is the only variety which can be discussed in these lectures because dropsy in the serous cavities can only be detected by special methods of investigation. Subcutaneous dropsy is recognised by pressing firmly on the skin for a few seconds with the finger. If the skin be healthy only a very slight depression is made by the finger, because the tissue beneath the skin is firm, and as soon as the finger is removed the depression disappears. On the other hand in dropsy the whole skin is stretched by the fluid underneath, and is therefore much swollen. The pressure of the finger pushes aside this fluid at one point, making a deep pit on the surface. When the finger is removed the pit remains for some time, because it takes some time for the fluid to return to the spot out of which it had been pressed. This sign is called "pitting on pressure." It is the one method of recognising the presence of subcutaneous dropsy, and nurses should be familiar with it. A dropsical limb or surface is pale, cooler than normal, and not so sensitive as normal.

Dropsy occurs under the following circumstances. First, in diseases of the valves of the heart, especially in disease of the mitral valve. It may also occur when the valves are healthy, but when the muscular wall of the heart is very weak, as in fatty degeneration of the heart. Also many persons who are dying of exhausting diseases, such as the malignant anæmias, consumption, diabetes, &c., get œdema of the feet towards the end; but in these cases, and also in bad cases of simple anæmia, the dropsy usually means that the muscle of the heart has become weak. In all the above cases the dropsy shows itself first in the ankles, then rises to the legs and thighs, and then fills up the peritoneal cavity (ascites) and pleura. It does not affect the face or arms unless some complication should set in. The next great cause of dropsy is disease of the kidneys, especially the varieties which are called acute inflammation of the kidney, the large white kidney, and the lardaceous kidney in its last stage. In all these cases the urine is scanty and contains much albumen. In kidney disease the dropsy usually begins in the face, which presents a very characteristic appearance; that is, it has a pasty look because the dropsy makes the skin pale, and a sleepy look because the eyelids are swollen. But it may also occur quite early in the labia majora of the vulva, and in the prepuce and scrotum. If patients with the above mentioned diseases of the kidney are able to walk about, the dropsy may occur in the legs, just as in heart disease; and if they are in bed and lie much on their back it accumulates in large quantity in the loins between the sacrum and the thorax, where it forms a large soft hump or pad. Finally, in bad cases, the dropsy may be all over the body, including the serous cavities.

When in charge of a case of heart disease, particularly of a mitral case, nurses should always be on the look-out for the appearance of dropsy. As has been said, it begins in the feet and legs and spreads upwards. If the patient is able to get up it will first be noticeable at night, and may have passed off before the doctor sees his patient in bed in the morning. Consequently nurses should look for it when putting the patient to bed at night, but should endeavour not to attract his attention while doing so. It is not necessary to formally press the skin with the finger so as to produce the characteristic pitting. It would attract less attention to casually hold the foot with the whole hand (on the pretence of feeling if it were warm, or on some other excuse) and allow the fingers to press firmly on the sides of the ankles for a few seconds, when any pitting would soon become evident. If the patient had been wearing a boot during the day its presence will have prevented the dropsy from occurring in the ankles, in which case pitting must be looked for in the leg, and in particular over the inner surface of the tibia. Sometimes the dropsy is revealed by a slight depression of the skin, caused by some pressure or constriction of the foot or leg, such as would be made by a wrinkle in the stocking or a prominence inside the slipper; or sometimes an obvious bulge may be seen at the top of the boot, where its pressure ceased. In such cases, of course, as the dropsy can be seen, it is not necessary to obtain the sign of "pitting on pressure."

When dropsy occurs towards the close of some debilitating disease, such as consumption, &c., it begins in the legs, and must be sought for and detected by the same methods as are use in heart disease. This remark applies also to the dropsy of anæmia, but sometimes that may be seen in the face.

On the other hand, in kidney disease the nurse must look for the onset of dropsy when she is washing and dressing her patient. The swelling of the eyelids is often most marked in the morning, and, curiously enough, if the patient has slept for a long time on one side the eyelid on that side is often very œdematous, while that on the other is scarcely affected. Nurses should also look for the "pad" in the lower part of the back, which has been mentioned. Also any difficulty or pain in passing water should be carefully reported to the doctor in the case of both sexes, for it may mean that the escape of the urine is greatly obstructed by swelling of the prepuce or vulva. Very great care should be taken in cleansing the vulva when it is much swollen by dropsy, because the irritation of the urine may produce some inflammation of it. The inner surface of the labia should therefore be washed with boracic lotion and then powdered after each micturition.

### To Nurses.

**WE invite contributions from any of our readers, and shall be glad to pay for "Notes on News from the Nursing World," or for articles describing nursing experiences, or dealing with any nursing question from an original point of view. The minimum payment for contributions is 5s., but we welcome interesting contributions of a column, or a page, in length. It may be added that notices of entertainments, presentations, and deaths are not paid for, but, of course, we are always glad to receive them. All rejected manuscripts are returned in due course, and all payments for manuscripts used are made as early as possible after the beginning of each quarter.**

### Nursing Europeans at Kota Kota, British Central Africa.

By a Correspondent.

In a former article I described nursing amongst the natives at Kota Kota. It may, perhaps, interest the readers of "The Hospital" *Nursing Mirror* to hear something about nursing amongst the Europeans.

When I first arrived there were only eight Europeans resident in the place. My first patient was a young Swiss trader, who was taken ill with hæmoglobinuric fever ten days after my arrival, and who sent for me at once. It was the first case of the kind that I had seen, and I did not feel particularly happy as I walked over to his store through the blazing midday sunshine. I found him just beginning his second rigor, temperature 103°, pulse 110, lying alone in his room, with his "boy" too fast asleep outside to hear his call. No extra blankets were forthcoming; hot bottles—so absolutely necessary in every African settler's house—were not to be had. I covered him up with all the garments I could find, and returned to my own quarters to collect all the necessary nursing appliances, and then took up my abode at the store for the time being. He was very delirious all the first night, and I had some difficulty in keeping him in bed. The room swarmed with mosquitoes, and rats ran boldly about the floor, and even climbed on to the table to see what they could get. Fortunately no complications occurred, and the hæmoglobinuria cleared up fairly quickly, so that after three days I was able to return to my own more comfortable dwelling, just visiting the patient daily till he was about again. Most people get attacks of ordinary malaria three or four times a year, and the treatment in these cases is quite simple—chiefly a matter of quinine medicinally, and of ordinary nursing and dieting. These attacks are generally over within a week; the patient feels very ill, and has a high temperature for two or three days, after which it comes down and he quickly convalesces.

### "Wanted to Die."

But all cases are not by any means of that simple character. On one occasion I was awakened at midnight by a loud knocking at my window, and a voice saying that some one at the store "wanted to die." It did not take long to dress, and I was soon on the way thither, trying to gather information from my guide *en route*. All I could learn was that the patient had fallen out of bed and "wanted to die." I found this description was fairly accurate; the patient was on the floor in a very violent fit, his teeth were firmly clenched, and all his muscles were quite rigid, and his pulse was quick and bounding. The only other white man living in the house had heard a loud noise about three-quarters of an hour before, and going to the patient's room had found him in this condition. He had complained of headache and sleepiness early in the evening, and had gone to bed as usual. We knew nothing of his previous history; the fit might have been epileptic as far as I could tell, so there was nothing to do but keep him from hurting himself. It lasted four hours, and then his muscles relaxed and he slept. He was unconscious all the next day, but swallowed fairly well, and on the day after he began to look about him and to talk; but he did not realise where he was, nor did he recognise anyone. This continued for some days; he seemed quite well bodily, but had no recollection of anything that had occurred during the last fortnight. As soon as a steamer came I sent him to the nearest doctor, who kindly wrote and told me that the patient had been suffering from cerebral malaria.

### Another Strange Case.

On another occasion a telegram arrived from a station thirty miles distant, asking if a sick man could be taken into the hospital, and the patient arrived without waiting for a reply. He was carried up from the shore in a hammock, and promptly fainted as he entered the door. It was only after some hours that I could get him to give any account of his illness, and then he was very vague about it. He had had fever a fortnight before out in the bush, and had got better; then three days ago he had thought it might be a good thing to take some quinine, so he had taken 15 grs., and had at once got much worse, and had telegraphed to us. This account did not help me much, but the patient was too exhausted to be worried about details, so I had to be satisfied with it. For two weeks I was not quite sure whether he would live or die. He hated food of any kind, and as nourishment was the one thing he wanted life was a perpetual battle. I sometimes spent half an hour in persuading him to take a small feed of milk. I was almost beginning to despair when I heard that a doctor was passing through on his way to Rhodesia; so I sent to him, telling him the state of affairs, and he came at once to see the patient. I shall always have a grateful recollection of that doctor, for he acted like a tonic, and I had not nearly so much trouble with the feeding afterwards. The patient stayed six weeks; he might have departed much earlier, but developed an imaginary eye disease, which he refused to have treated, and which made it impossible to go anywhere else. I got an attack of fever, so that he had to go; but before leaving he came to tell me that he had discovered that he was suffering from conjunctivitis, and that the proper remedy for that was nitrate of silver, and that he should like some to take with him. Of course, I did not give him any.

### A Doctor Made Deaf by Quinine.

One of my patients was a doctor who had come into the country to study malaria. He had a slight attack of hæmoglobinuric fever while at Kota Kota, and sent to ask me to make him up some medicine and to go and see him. When I arrived he addressed me in such stentorian tones that I thought at first he had cerebral symptoms, and was much relieved to find that it was only because he was deaf from large doses of quinine. I went to see him daily, and he always persisted in opening the door; and when I protested that he ought to be in bed, he would at once lie down on his bed and say, "So I am." He afterwards assured everyone that he had been a most exemplary patient.

### Touching Faith in a Tincture.

My last serious case was that of a man who possessed a profound and touching faith in Warburg's tincture. When he felt feverish he took a large dose of that famous drug, piled on blankets and hot bottles, got into a profuse perspiration, and was all right the next day. He told me this so often when he was in good health that I quite began to wish he might have a little fever, and give me a practical demonstration of how he treated himself. I was rather surprised, therefore, to get a note one morning asking me to come and see him. I found him seated in a chair in a strong draught, with a temperature of 101° Fahr. The Warburg had been taken, but, contrary to expectation, had not acted like a charm, and he was feeling very ill indeed. This was hardly astonishing, since he admitted that the perspiration had made him sticky, so he had taken a bath for the sake of comfort. He submitted to be treated in a rational manner for a few days, and apparently got quite well. I was shortly afterwards called away to another part of the Lake, and did not see him again, but I heard that he had suffered a relapse, which, I imagine, he treated in his original manner, and became so much alarmed at his state of health that he obtained leave of absence and went home.

### The Nurses' Co-operation.

DANGERS AHEAD.

The twentieth annual report of The Nurses' Co-operation, the most successful organisation of its kind, is not satisfactory from a purely business point of view. It shows that the whole of the reserve funds of the Co-operation have been sunk in the Howard de Walden Nurses' Home and Club. This club may be regarded as an experiment, and for the year ending December 31st, 1900, according to the published accounts, the expenditure in connection with the working of the club exceeded the receipts by nearly £200. In addition to this expenditure it is essential, as the premises are leasehold, that a sinking fund should be at once set up which will repay the capital outlay well within the period of the lease. It further appears from the report that the secretary is a member of the committee of management, and that the committee of management as at present constituted, so far as the members' representatives are concerned, is re-elected year by year practically without change. The funds to be dealt with are very large, exceeding as they do £40,000 a year, which renders it essential for the protection of the nurses' interests that the best business ability shall be brought to bear upon the management of the finances of the Co-operation, and we are of opinion that the committee should be strengthened in this respect at once as a safeguard for the nurses who have joined the Co-operation. We know of no other business where a paid officer is a member of the committee of mangement, nor where such large funds are dealt with without the committee containing at least two men of business, of position and experience. It would be a grave calamity should anything happen to imperil the financial position of this greatest of nurses' co-operations, and we would earnestly ask the nurses on the staff to put their heads together, and through their representatives on the committee, to move in the direction of securing the introduction of fresh blood by the nomination of a larger number of members, such members being selected from amongst such friends of the nurses as possess business capacities which entitle them to implicit confidence. If each of the staff nurses, or the majority of them, were to set themselves to induce one such person to become a member of the Co-operation, they would take the step best calculated to secure the permanence and financial strength of the Nurses' Co-operation. In any case, it is essential to introduce new blood each year on the committee of management, not only through the nurses' representatives, but by the regular retirement of the nominees of the members and their replacement by new and energetic people of capacity and judgment. As matters stand at present, a little loss of popularity, a slight falling off in the earnings of the nurses, and the Co-operation may be face to face with a deficiency which it may be difficult to provide for, and which may even endanger the safety and permanence of the whole institution.

### Minor Appointments.

Maidenhead Infirmary.—Miss Ada M. Bates has been appointed assistant nurse. She was trained by the Meath Workhouse Nursing Association at St. Peter's Home, Kilburn.

Royal Boscombe and West Hants Hospital.—Miss Annie Brown and Miss Daisy Preece have been appointed staff nurses. Miss Brown was trained at the Seamen's Hospital, Greenwich, and the Women's Hospital, Soho, and Miss Preece at the General Hospital, Wolverhampton.

Royal Bath Hospital, Harrogate.—Miss Drummond Sadler has been appointed head nurse. She was trained at Edinburgh Children's Hospital, and has since been sister at York County Hospital for three years, and sister at Cheltenham Hospital for six years. She has also done some district and private nursing.

Royal Devon and Exeter Hospital.—Miss Rosa J. Hockley has been appointed sister. She was trained at Guy's Hospital, and holds the L.O.S. certificate. She has been, in succession, nurse for six months at the Western Hospital of the Metropolitan Asylums Board; head nurse in the private nursing home at Guy's; head nurse in the gynæcological department; and temporary sister at Guy's Hospital.

Colchester Hospital.—Miss Florence E. Tylecote has been appointed night sister, and Miss Constance Blackles day sister. Miss Tylecote was trained at Cheltenham General Hospital, and has since been sister in the same institution; assistant nurse at Chelsea Hospital for Women, and sister in charge of the female block at the Central London Sick Asylum, Hendon. Miss Blackles was trained at the South Devon Hospital for three years, and has since been on the private nursing staff of the same institution.

North London Consumption Hospital.—Miss Sadie MacCormac has been appointed night sister. She was trained at the Radcliffe Infirmary, Oxford. She was next sister for two years of the women's medical ward at the Birmingham Infirmary, and then did private nursing for the Hollond Nursing Institution in Paris and the Riviera for the winter months, subsequently acting as holiday sister for four months at Brompton Chest Hospital, and sister for one year of the men's medical ward, with charge of the theatre, at Paddington Infirmary.

### Presentations.

Chelsea Infirmary.—The nurses at Chelsea Infirmary are deeply regretting the loss of Sisters McKay and Rougier, who have both resigned their appointments. In the words of one of the staff, Sisters McKay and Rougier will be very much missed, "for they have done everything to make us feel that indeed we have a veritable home in Chelsea Infirmary." Sisters and nurses have combined to give Miss McKay a pretty clock, similar to the one presented to Miss Rougier; and Miss Barton, the new matron, presented her with a writing case, with silver monogram. Other gifts to Miss McKay, who has won the friendship of many during her six years at Chelsea, included a silver frame from some of the women patients, and a silver toast rack from the "scrubbers."

Eccles Technical Classes.—At the close of a very successful course of lectures on "Sick Nursing" given at Eccles in connection with the Eccles Technical Classes. Mrs. Elsie French, of the Higher Broughton "Nursing Home," Manchester, was presented by her pupils with a very useful carriage clock in case, a beautifully embossed silver plant pot cover, and large silver and cut glass smelling salts, and all expressed a hope that she would continue the lectures next session.

Harlech and District Nursing Association.—Nurse Lewis has been presented with a handsome dressing-case by the committee and grateful patients upon leaving her post in connection with this association.

### Novelties for Nurses.

A SERVICEABLE WATCH.

A bad watch is worse than none, as all those who have suffered from one that is unreliable know. Some time ago we introduced to our readers' notice a watch which, after giving it a fair trial, we found that we could safely recommend. This is a watch which Messrs. Hemings, of Conduit Street, sell for £2 2s. in silver. We have recently had one of these watches in our possession for the purpose of testing, and are glad to say that our second experience quite bears out the high opinion we had formed of the reliability of the little watch. Two guineas is a moderate price to pay for a really good watch, and when we inform nurses that no extra charge is made for the engraving of initials, we are sure they will realise that here is a suitable object for a presentation.

### A Personal Experience in District Nursing.

A correspondent sends us the following account of her early experiences in starting as a district nurse:—

"What we want," said the doctor, "is a district Nurse!"

Some years before, the need of district nursing in an isolated northern district had impressed me; and, seeing that the supply by no means equalled the demand, I had always wished to materially increase that supply, even by inducing nurses to work more for love than for money, where they could. The doctor's remark gave me an idea, and the next morning I called on Canon ——, told him what the doctor said, and offered to beg during one month, start the work, and go on with it as long as funds permitted, or till it was established.

Canon —— cared much for his sick poor, and I was well known to him. So he wrote a letter, authorising me to beg of our own people, blessed me and my undertaking, and dismissed me.

### A Month of Begging.

Oh, that month of begging! Some sympathised deeply, but really the calls were so many! Some would think of it—and that was all they did. Rich ladies were deeply interested, and requested me to call on them. Then hope revived, but I was disappointed. One damp and chilly morning in late October I called on a tradesman in his shop and handed him the Canon's letter, in nervous haste to explain my errand. He "knew nothing about me; he would make enquiries and then see!" That same afternoon, muddy and sad, having trudged four miles and lost my way in many lanes, I went to beg of Lord —— at his lovely old hall. Cordial sympathy, a perfect understanding of my motives, and a £5 donation sent me away consoled! When such as he "felt out" my scheme, as it were, they helped me morally as well as materially. But the undreamt-of suspicion of commercial enterprise, which seems generally inherent in commercial minds, sometimes wet-blanketed my enthusiasm nearly to extinction.

### My Headquarters.

Meantime, I called on the parish doctors, explaining my intentions, and leaving addressed postcards. Also I took a garret room, divided into two by a wooden partition. Slanting walls and ceiling were dirty whitewash. Floor and windows were grimy. The fireplace, built out into the room, was an unsightly shapeless mass. Lumber and cobwebs were everywhere. The godmother who, like all my relatives, thought me a fool, was yet my loyal helper; and she undertook to have that room made habitable—to furnish it, and to have all comfortable by the day I started work. Lastly, I asked for a treasurer, and placed money and accounts in his hands.

### The First Round.

It was a hopelessly wet November morning when I started on my first round. Swish, swish, went my feet through the unpaved streets of the slums. Soon all the starch was gone out of gown and bonnet-strings—white apron and front hair were damp, and I felt limp and chilly. But a vision of my tiny flat, furnished, curtained and warm, a picnic meal, and my godmother's company, kept up my spirits. A little before one o'clock, as I climbed the stairs, dripping, tired, and hungry, courage came with each step up. I opened the door and looked in.

There was a bright fire, without fender or fire-irons. Before it, on a towel-horse, hung a new pair of stockings and a pair of large list slippers. Nothing more—and the wet floor had evidently just been washed. I looked for a dry place where I might sit down and cry.

A step creaked on the stairs, and my godmother came in, wet and beaming, with a basket full of paper bags and parcels in her hand.

"My poor, dear child, how wet you are! When I came here, an hour ago, I found nothing in the room except the woman who was scrubbing it, and her pail. So I had a big fire made, bought those stockings and shoes because of my rheumatism, and then went out to purchase food. Now we must just sit over the fire and wait till that wretched man brings the furniture."

### Two Chairs and a Floor.

I went down and borrowed two chairs, and what else was necessary, and while the tea brewed we sat one on each side of the fenderless fireplace, the while from us, from the floor, and from the garments on the towel-horse, there went up a steaming dampness. My godmother's face was tragic.

"My dear," she said with solemnity, "you certainly *will* catch your death!"

But the smell of tea had cured all my sadness.

"*Do* pour it out!" I implored. "What fun we're having."

"I don't see much *fun* in sitting on hard chairs in a wet, empty attic!" she answered with severity.

The floor for a table, when one's seat is a high chair, is inconvenient. However, we managed to eat and drink a good deal. It was growing dusk when the furniture came at last, and in the midst of our bustle over arranging it a doctor came about a case. This was encouraging. In spite of the almost tearful warnings of my godmother, I slept that night in my flat. I have a feeling that if you undertake to do a given thing at a given time, and then let trifles prevent you, you get a bad start, and don't prosper.

### Work Comes In.

After my former experience of district nursing, I had only to model the new routine from the old, with regard to work, book-keeping, recreation, and other details. Work came in, thanks chiefly to my doctor friend, and, besides that, it is astonishing how soon the existence of a district nurse becomes known. I believe that any trained nurse who started a district in a slum would always find work enough, and success enough, to content her with her undertaking, provided that she was loyal to the doctors and tactful with the patients. It is five years now since I began: financial gaps have been made good by the Canon, and I cannot says that the funds are, or ever have been, satisfactory; but the work lives and grows, and this year, by means of a big "Fête," opened by a speech on the necessity and uses of district nursing, we hope to make some progress.

### An Antidote to Despair.

The great damper on any enterprise of this sort is the suspicion, that is sure to be shown by many, of a self-seeking motive. It takes long to live down, and in some minds appears to endure to the end. Then there may come to the nurse phases of doubt of herself—of her fitness to succeed; and if, after all, it is worth while. To accept a vacant post, and fill it honestly, as a means of livelihood, is felt to be a sensible and safe proceeding. To step into a vacancy, approved as such by the silence and inaction of the majority, and to fill it, to your own loss and risk, is quite another thing. Common sense comes to the rescue, however. Every bit of work done would certainly not have been done but for the nurse. That thought is a grand antidote to despair.

### Practical Difficulties.

Another disadvantage of quite independent work is having no one at the back. For jealous and exacting patients, who cannot bear to have less attention than new and urgent cases, someone to refer them to is a great blessing. The only thing I could do was to explain the case to Canon ——, or to any doctor who might have the care of the case, and let the rest alone. Then to judge how much one can, or ought to do, is sometimes a burden on one's conscience. I come home late at night, and am greeted with a summons from a chronic case, where I've never yet found anything to do, and am morally certain that it will be the same to-night. But there is a possibility of danger. So I go; and when I get to the house it is locked up and the lights are out! No matron would have allowed me go, I know but conscience is a rigorous mistress!

THE OTHER SIDE.

So much for typical difficulties. Against them I place, next to the joy of knowing that what one does would otherwise be undone, the advantages of perfect freedom to do work when and where and how one feels it to be best at the moment. Above all, there is the thought that a work is being started that shall outlive oneself. If I get through the rough beginning, my successor will begin where I leave off. The suspicions I live down will not sting her; the doubts whether my work not answer will not come to her; the precedent of my plan of work will spare hers from complaint. So a meaning and a use is woven through all difficulty of starting, and none of it shall be wasted.

### Queen's Commemoration Fund on Behalf of Queen Victoria's Jubilee Institute for Nurses.

BY kind permission of Lord and Lady Londonderry the annual meeting of the subscribers to the Queen's Commemoration Fund was held at Londonderry House on Wednesday afternoon.

The Duke of Portland was in the chair, and was supported by the Hon. Mr. Sydney Holland (Treasurer) and Mr. Harold Boulton (Secretary). Among those present were Lady Londonderry, Lady Victoria Buxton, Sir Squire Bancroft, the Rev. Dr. Adler, and other well-known supporters of philanthropic institutions. Mr. Harold Boulton read the replies received, on behalf of His Majesty the King, to the letters, one from the Q.V.J.I., and the other from the Council and Committee of the Queen's Commemoration Fund.

The Duke of Portland, in alluding to the great loss sustained by the nation in the death of Queen Victoria, said by none was her loss more felt than by those who supported this Fund. There were few better instances of the continuity of character which she always showed than her decision to devote the money collected by the women of England to the object of providing nurses for the poor in their own homes. He had been asked to occupy the place of the late Duke of Westminster as Chairman of the Commemoration Fund, and he felt it to be a very great honour to be associated with such a work. He was glad to mark his connection with it by noting the fact that there had been an increase in annual subscriptions; more money was, however, wanted, and an appeal would shortly be issued, of a wider and more general nature than had yet been made; he hoped it would not be in vain. He then explained, in case any confusion existed in people's minds, the distinction between the Queen's Commemoration Fund and the Q.V.J.I. The Jubilee Institute had been started by Her late Majesty in 1887; the good work grew apace, and outstripped the original funds; the Commemoration Fund had therefore been started by the late Duke of Westminster and other practical philanthropists to render assistance to the Council, and to help in raising funds to carry on the work. The committee was composed of some outside the nursing world, as well as those more directly connected with it, and would, it was hoped, appeal to all classes and creeds. The Q.V.J.I. had now a palatial home in Liverpool; the Queen's nurses were at work in London slums, among the miners in Scotland, and in wild Achill Island off the coast of Ireland. The late Queen's visit to Ireland last year had given the work there a great stimulus. If only funds were forthcoming, much more could be done; how was the money to be found? That was the question, and it was one which a sub-committee were going to discuss at the close of this meeting. He trusted they would come to some decision, and that an appeal of a national character would be issued in the course of a few days. As at present constituted the Fund would have to go on appealing to the generosity of the public from year to year.

The Hon. Sydney Holland seconded the adoption of the report and balance-sheet proposed by the Chairman. The most important point in the balance-sheet, he said, was that £550 had been spent during the year in appeals for money. It was very difficult to bring out an effective appeal, and as personal appeals always seemed to be most effective, 44,000 personal letters had been written. The result was, he thought, a miserable one; £1,100 in donations and £1,000 in subscriptions; the latter might last perhaps three or four years, and then natural shrinkage would take place. "Ladies and gentlemen," continued the Treasurer, "the appeal *must* succeed. We cannot possibly allow a work started by the noblest woman who ever lived to end with her death; it is a most sacred trust. She loved the work; she loved the nurses who were doing it." Mr. Holland then spoke of the visit of the Queen's nurses to Windsor, which was impressed upon his own mind by the fact that he had had to teach them all to curtsey; how she had at first intended only to drive past, but eventually went among them, and said, "Nurses, I thank you for the work you have done, and the work I know you will do." Was the work to end for the want of money? The Medical Officer of Penzance had said that he could always tell in which streets the Queen's nurse had been at work by the improved hygienic condition of those streets. The Queen had insisted on the nurses being trained. To show the necessity of training he quoted a "true story" which he had heard a few days ago: a country nurse, hearing that the doctor had said a poor woman suffering from appendicitis was to stay in bed, had urged the patient to get up, as staying in bed would make her so weak. The doctor further ordered milk and soda, and this nurse gave washing soda in the milk! £500,000 were needed, but a million would not be amiss. There should be a nurse in every town, in proportion to population; a midwife should work under the nurse and there should be district nurses in the villages.

Other speakers were Mr. F. D Mocatta, Mr. Brinsley Marlay, General Prendergast, and Monsieur Janvier, *Chargé d'Affaires* of Hayti, who said that, when a doctor in Paris, before he was called to take part in the work of diplomacy, he had worked with nurses; he knew their devotedness to duty, and their genuine goodness of heart.

A vote of thanks to Lord Portland and to Lord and Lady Londonderry brought the meeting to a close.

### Echoes from the Outside World.

AN OPEN LETTER TO A HOSPITAL NURSE.

It appears that preparations are in train to offer two rare gifts for King Edward's acceptance. One is the suit of armour which was worn by the champion Dymoke at the last Coronation. The armour is of steel, richly inlaid with gold, and is valued at £4,000, which sum is to be subscribed by forty gentlemen, each giving a hundred pounds. I suppose the idea is that the King would like the champion at his own Coronation ceremony to use the same armour as that worn when his mother became Queen. The other gift is likewise intended to be of use on the day when the King wears his crown for the first time, and it is especially interesting just now because it is offered by Mr. Maurice Lyons, one of Australia's richest squatters and most successful lawyers. It is a very splendid opal, one of the largest ever seen, weighing 250 carats, and measuring two inches long and an inch and a-half deep. It was found about six years ago in the Opalton district of Western Queensland, and a large piece, almost half as large again, was broken off in the removal, or its size would have been yet more extraordinary. It is to be offered to the King as a contribution to the Crown regalia, and as the Royal Family have no qualms about opals being "unlucky," her late Majesty having frequently given away opals as presents to relations and friends, it is hoped that the King will further gratify the colonies by thus allowing them to share in providing the jewels for the Crown.

On Saturday the Duke and Duchess of Cornwall and York leave our shores on their long Colonial trip. Naturally our best wishes go with them, that they may have a prosperous and safe voyage, a pleasant health-giving time—much of it will hardly be of the nature of a holiday—and that their appearance amongst our brothers and sisters of far-off lands may tend to the true cementing of the bond which binds us all together. As far as human ingenuity and human labour can go, everything which can make for safety has been done on board the *Ophir*. She is so divided by watertight bulkheads that she will float in safety with any two compartments thrown open to the sea, and she has a cellular double bottom, further divided into water-tight compartments. Though not the most important vessel in the Orient fleet, she has the same horse-power as if she were the largest, and her two independent triple engines will drive her at the highest speed possible with comfort. The size of the vessel may be best realised when one remembers that besides the Duke, the Duchess, the Royal suite, and the Royal servants, there will be 27 officers on board, 125 bluejackets, 100 marines, 37 bandsmen, 20 boys, 7 engineer officers with 88 men under them, a purser, 50 stewards, a Royal chef specially engaged, 9 cooks, 3 bakers, 2 butchers, 1 printer, 2 barbers, and a laundry man and his wife. As for the Royal apartments, though very spacious and comfortable, the chief aim of those who have remodelled the vessel has evidently been to make the floating house as much as possible homelike in character, and the quiet simplicity of the decorations and the introduction of pictures, portraits, and knick-knacks from York House has done much to attain this object. Those on board bear testimony that the Duke and Duchess have themselves made many of the arrangements and from the first have taken a special interest in all the details. The number of pianos in the sitting-rooms is especially remarkable as also the preparations for private theatricals at the end of the large dining saloon. One end of this splendid apartment forms a natural proscenium and a portable stage was amongst the baggage sent on board.

The private apartments of the Royal pair are of the greatest interest, though all the others are so perfectly arranged that it is difficult to pass them over altogether. The dining-room has a minstrel's gallery, and the electric lights are placed between the dome of stained glass and the outer roof so as to shed a subdued light over all. In place of the revolving seats which are usually provided for the saloons on board ship are Chippendale chairs with a very wide base and upholstered in sealing-wax red leather. The drawing-room has a most beautifully soft carpet upon which it would be difficult to slip even in very rough weather, and has rosewood and satinwood panelling with Sheraton furniture. In the smoking room the armchairs are of dark green leather and oak, and little card tables and ingenious writing tables are scattered about here and there. The bedrooms both have swing cots, which should prevent much motion being felt, and over the Duke's bed is a portrait of the Duchess and another of Queen Alexandra and Prince Edward of York, beneath which is written "Grannie and Baby." In the Duchess's room, which is all white, there is a large clock, the face of which can be illuminated by her from her bed by the electric light at a moment's notice. Her boudoir is in a subdued tone of green, and is very tasteful. Every room is warmed by heating apparatus, and can be cooled when required by a bee's-wing fan in the centre of the ceiling worked by electricity. Amongst the other most noticeable pictures is a group, beneath which is written in a feeble hand "V. R. I. and grandchildren of York, Osborne, August, 1900"; a portrait of Lord Roberts, and a representation of the Irish regiments fighting at Pieter's Hill.

The appointment of the Bishop of Stepney to the See of London is an event of special interest to the nurses in the East-End of the Metropolis who in the pursuit of their avocation have so often come across Dr. Winnington Ingram, and know, by personal experience, something of his wonderful influence amongst the masses. They can confirm the testimony which is forthcoming from all quarters of his power of kindling enthusiasm, and they are not ignorant of the secret of it. It is true that the successor to Dr. Creighton is not without the qualities which charm those who never look below the surface. Dr. Winnington Ingram is an original, a forcible, and an eloquent preacher. His sermons hold the attention of a fashionable West-End congregation, as well as that of the sons of toil. In spite of all the claims upon his time he has never allowed his discourses to be slovenly; they do not lack finish. His accessibility and pleasant unconventional manner have won him many admirers. But the principal reason why people to some extent familiar with the labours of the Bishop-Designate of London, are delighted with the appointment is because of the life of the man. It is a life that ever since he took up his abode at Bethnal Green has been obviously lived in the service of the Master. Of the poor and the oppressed he has been the unfaltering champion; he has taken by storm the hearts of the rough; he has been the means of converting scoundrels into saints; he has brought sunshine into slums, restored happiness to desolated homes, has made himself the friend of defenceless women and forlorn children. In a wider sphere of activity, with splendid opportunities for zeal and faithfulness, it is felt in East and West alike, that, animated by the same spirit which has so far characterised his career, he will not merely achieve fresh distinction but will also strengthen the hold of the Church upon the affection aud the respect of the community.

### Everybody's Opinion.

[Correspondence on all subjects is invited, but we cannot in any way be responsible for the opinions expressed by our correspondents. No communication can be entertained if the name and address of the correspondent is not given, as a guarantee of good faith but not necessarily for publication, or unless one side of the paper only is written on.]

### A WARNING.

"MATRON" writes: On the evening of January 26th a man giving his name as Alfred Miller came to the Hinckley Cottage Hospital saying he had taken 1 oz. of nitric acid instead of whisky and gave up a small flat bottle containing nitric acid. He seemed in great pain and was detained a few days. On leaving, the sum of 6s. (which was collected amongst the staff) was given him, as he said he was out of employment. We are quite sure that he is the same man who has gained admittance to other hospitals with the same tale.

"THE MATRON of the Cottage Hospital, Brentford," writes: A young man, giving the name of Alfred Churchill, and age 23, was admitted to this hospital on the 16th of last November, under exactly similar circumstances to those described. He was under treatment for five days, and feeling sorry for his apparent destitution, we gave him a small sum for railway fare and lodging.

### THE UNIFORM QUESTION.

"A TRAINED NURSE" writes: There has been dissatisfaction for some time among nurses *re* the use of uniform among a class of people who are not, and never would be, members of our profession. I may mention as a fact that only three doors from where I am nursing a general servant, when she goes out, appears in the uniform of a trained nurse, with gossamer and white strings. Would it not be possible for this to be stopped, by everyone who wears uniform being compelled to show a certificate that they are *bonâ-fide* nurses?

### TWO CONCEPTIONS OF THE NURSING VOCATION.

"HUMILITY" writes: I have read with interest the letters on this subject, and hope you will find room for another in your paper. I consider the nursing profession should be as "strictly religious" as the ministerial one. A nurse certainly does not require to "make a parade of piety." Her "religion" is shown in the way she does her work and her manner towards her patients. Like a "Matron of Experience," I, too, have a large personal acquaintance with the clergy, and I have never heard a nurse spoken of by any of them except in the highest terms of esteem. A great many of us owe our present posts to the good opinion a clergyman has of us. I do not think a "Matron of Experience" has quite grasped the good rector's meaning. Some families do treat their nurse as one of the family, but a nurse cannot always expect to be so treated. A nurse's mode of living must, of necessity, be frequently changed so as to suit her employers—not herself. I think most nurses would rather dine with the housekeeper (who is generally a lady of as good a standing as a nurse) than in a large dining-room all alone. The nurse who could not stay with a patient on account of her place of dining would do the patient no harm, but may have done herself a little harm. It is doubtful if even a "Matron of Experience" would care to employ on her staff one who thought herself above dining with a housekeeper or in a housekeeper's room.

"COMMON SENSE" writes: The "Experienced Matron" who has permitted herself to write with such discourteous contempt of clerical and conventional life should beware how she encourages the unfortunate tendency to undue self-importance, among members of her own profession by upholding the petty self-assertion of the nurse who resigned her duty rather than risk her dignity by dining in the housekeeper's room at a large London hotel. The inference that a gentleman who could suggest this apparently simple course "would not be a safe patient for any honest woman to nurse" is curious! One wonders by what train of profound "logic" the "Experienced Matron" evolves a human monster out of such very slight material! She must be very far-seeing! Would she consider it quite safe and orthodox for a nurse to dine at *table d'hôte* without chaperone or escort? In her anxiety to uphold the social status of lady nurses she mentions that "many of them are accustomed to dine late, and when not dining out, with possibly the aristocracy, that they would always prefer not to dine with the family of their patient." Surely these little arrangements should be left to the discretion of the lady of the house The experience of many is that the professional nurse too often becomes self-important—possibly from the techical knowledge and authority of the position—and that she would do well to cultivate the gentleness, patience, and sympathy, as well as skill, so requisite in the noble work of nursing the sick, irrespective of class.

"A WARNING VOICE" writes: I have read with some bewilderment the letter from "A Matron of Experience" under the above heading. It seems to me that the question really at issue is not whether a nurse is or can be classed as a nun or a demi-mondaine (!) but one that is always cropping up under various forms—viz., What is a nurse's social position? Now it would be very much better, and it would save a great deal of heartburning, if we nurses could drop the fiction that "every nurse is a lady" and face the truth that, apart from her own position in society, a nurse has no social status by virtue of her profession. She has, however, what is far better, the respect and esteem of all as a member of a useful and honourable calling. How can it be otherwise? We all know the nursing profession is very "mixed." It "rose from the ranks," so to speak, and the immortal "Sairy Gamp" and "Betsy Prig" were typical exponents of the art of nursing in its early years. Nowadays, though daughters of men in both services and in the various professions become nurses, the greater number are recruited from the middle and lower-middle classes. And the "mixture" is a good thing, for employment can thus be found for all classes, in the varied duties and positions of village midwife, asylum attendant, parish, workhouse, district, private, and hospital nurses, army, navy, and hospital sisters, matrons, and lady superintendents of large institutions, and of the army and naval nursing services. It would be a very great pity if we nurses were to lose the *substance* of respect and esteem our calling ensures us in the vain pursuit of the *shadow* of social position, which by its very constitution our profession has not the power to give.

### "ONE OF THE GREATEST NEEDS OF THE DAY."

"A MERE OUTSIDER" writes: Will you allow one who is not a member of the profession, but has seen a good deal of nurses, a little space in your columns to point out what to many middle-class families is one of the greatest needs of the day? The "lady-nurse," who "is in the habit of dining late as a matter of course,"[1] and when off duty with the aristocracy, is quite out of place in their humble abodes. Except in very rich and luxurious homes illness is apt to bring with it discomfort for most members of the household. Meals become irregular, the demands of the sick-room necessitate some lightening of the servants' work in other directions, and all the family must be prepared to put up with some discomfort and lend a hand here and there as required. Under such circumstances the large demands made for her own comfort by the "lady-nurse" are the "last straw" which completes the general disorganisation. We are willing to leave these luxuries to those who can afford them. What we require is some trained women of a lower class who can make themselves generally useful to the patient without loss of dignity. Many lady's maids and housemaids—even some general servants—make capital nurses, and their previous training has generally taught them how to prepare appetising dishes and to move softly about a room—two arts which seem almost unknown to the average nurse. Is it not possible to arrange in some way for the training and organising of a second grade of nurses more suitable for the needs of persons with an income of less than £500 a year?

[1] *Vide* "Nursing Mirror" of March 2nd.

### Appointments.

Portsmouth Workhouse Infirmary, Milton.—Miss Jessie Hall has been appointed matron and Miss Hannah Goode has been appointed assistant matron. Miss Hall was trained at Birmingham Infirmary, where she has since been ward sister, home sister, and assistant matron. Miss Goode was trained at St. Pancras Infirmary. She has since been staff nurse and night superintendent in the same institution, and charge nurse at the Brook Hospital, Shooter's Hill.

Newbury District Hospital.—Miss M. A. Stonehewer has been appointed matron. She was trained at the Royal Albert Edward Infirmary, Wigan, where she has since been sister in charge. Subsequently, she has been sister and deputy matron at the Royal Bucks Hospital, Aylesbury.

Chelsea Infirmary.—Miss Alice Mutter has been appointed assistant matron. She was trained at St. Mary's Hospital, Paddington, and since 1898 has been charge nurse at the Western Hospital, Fulham. She holds the L.O.S. certificate. Miss Emily Legat has been appointed home superintendent. She was trained at the Royal Hospital for Sick Children, Edinburgh, and St. George's Hospital, London. She has since been night nurse at the Royal Hospital for Sick Children, Edinburgh.

Northern Convalescent Fever Hospital, Winchmore Hill.—Miss Edith Firth has been appointed assistant matron. She was trained at Mill Road Infirmary, Liverpool, and has since been night superintendent at the Northern Fever Hospital, Winchmore Hill, and charge nurse at the Park Hospital.

### "The Hospital" Convalescent Fund.

The hon. secretary acknowledges with thanks the receipt of 2s. 6d. from Miss E. Lucas.

### Wants and Workers.

Nurse Greaves, Union Infirmary, Old Chesterton, Cambs, would be very grateful for presents of infants' clothing for children from six months old to enable young mothers to start again in life.

### TRAVEL NOTES AND QUERIES.

Grindelwald in May (Yungfrau).—I cannot get you as yet very definite informatio 1 about Swiss tours: they will not be compiled for some few weeks yet, but I will not forget you, and if you cannot wait write to me again. If possible do not go till the first week of June. May is full early on account of the flowers, and the passes will not be open.

Books Relating to North Italy (Cam).—The best book on Rome is Hare's "Walks in Rome": it is portable in two small vols. His "Cities of Northern and Central Italy will give you charming accounts of Milan, Venice, Florence, and Genoa. This work is voluminous in four vols. and only suitable for home consumption. There is a valuable book of a lighter sort by Mrs. Minte Elliot called "Roman Gossip," and be sure to read Hawthorne's "Transformation" and Ouida's "Pascarel" and "Ariadne." I am writing on Rome now, and in the issues of December 1st, 15th, and 29th, with January 19th you will find the first four papers. For guide books Baedeker is the best; Murray is excellent from an æsthetic point of view, but he is not so commonplace and practical. It is always a pleasure to help anyone so appreciate as you are.

Tour in Brittany (Robertson).—It depends greatly on where you want to go, whether the sum you have at your disposal is sufficient or not; if you remain quite in the north, or go directly to the south; but both cannot be done. I fear you are allowing yourself rather a small margin; 35 frs. is only £1 8s. 4d.—that is, 4s. a day. If you are experienced travellers, which I fear you are not, you might get board and lodging for that; but how about other expenses, small excursions, tipping, washing, &c.? For £10 for a fortnight you might do as follows:—Second-class return to St. Malo, £2 1s. 2d. Straight on to Mont St. Michel viâ Dol (try to do it on a Saturday to see the typical market at Dol), stay till Monday, then train to St. Brieuc. Stop there one night and take diligence to drive round to Paneipol and see the fine coast. The diligence there meets another to Lannion, where stop as long as you like and there rail to Morlaix. This last is a very fine old town full of picturesque interest. If you prefer south you might visit Quimper, Quimpelé, and Aurey for about the same sum, and a third alternative is confining yourselves to the Bay of St. Malo, making St. Servan your headquarters. You have plenty of time to let me know which of these arrangements you prefer and how I can help you further.

### For Reading to the Sick.

"WORD OF CONSOLATION."

Lord, Thy Word abideth,
And our footsteps guideth;
Who its truth believeth
Light and joy receiveth.

When our foes are near us,
Then Thy Word doth cheer us,
Word of consolation,
Message of salvation.

When the storms are o'er us,
And dark clouds before us,
Then its light directeth
And our way protecteth.

Word of mercy, giving
Succour to the living;
Word of life, supplying
Comfort to the dying!

*Hymns Ancient and Modern*, 243.

A French writer—Coppée—the exquisite taste and purity of whose style has long been the admiration of Europe, may tell us of the satisfaction that is found in the simplicity of the Gospel. He was lying for days motionless at the end of a long and dangerous illness, and the sustenance of his weary frame was this: "For weeks and months, passed in my bed and in my room, I lived with the Gospel, and little by little each line of the Holy Book became alive for me, and assured me that it spoke the truth. Yes; in all the words of the Gospel I have *seen* the truth shine like a star, I have *felt* it like the beating of a heart. How could I any longer hesitate to believe in miracle and mystery when there had happened within me a transformation so profound and so mysterious? For my soul had been blind in the light of the faith, and now saw it in all its splendour; had been deaf to the word of God, and heard it to-day in the sweetness of its persuasion; had been palsied with indifference, and now raised itself to heaven with free wings, and the foul demons that had possessed it were cast out for ever. . . . So the words of a humble Workman of Galilee, entrusted by Him to some poor men with the command to teach the nations, resound still, after nineteen centuries, wherever man is no more a barbarian." These are the words of genuine experience from a master in literature.—*From "Happy Suffering."*

The deep dark shades may whelm the day,
And all the splendours melt away;
The night may lower—but not for one
Whose life is hid beyond the sun;
My God shall make the darkness bright,
"At evening time it shall be light."

It shall be light; and all below
My soul believed in, it shall know;
Unclouded then mine eyes shall see
The heart of every mystery:
In all Creation's depth and height
"At evening time it shall be light."

It shall be light; when I behold
The Blessed Vision long foretold!
The dearest hope, the sweetest grace—
My soul's Belovèd face to face.
Dear Lord, upon my longing sight
Oh, bring the evening and the light!

—*Rev. S. J. Stone.*

### Notes and Queries.

**The Editor is always willing to answer in this column, without any fee, all reasonable questions, as soon as possible.**

**But the following rules must be carefully observed :—**

**1. Every communication must be accompanied by the name and address of the writer.**

**2. The question must always bear upon nursing, directly or indirectly.**

**If an answer is required by letter a fee of half-a-crown must be enclosed with the note containing the inquiry.**

*Plague at Capetown.*

(259) I am anxious to volunteer to nurse plague at Capetown. Will you kindly let me know to whom I should apply ?—*Sphinx.*

We do not think that any nurses are being sent out, but you might apply to the Colonial Nursing Association, Imperial Institute, S.W.

*Asylum at Capetown.*

(260) Would you kindly tell me to whom I can apply for vacancies in the Asylum mentioned in the issue of February 16th ?—*Nurse R.*

Write to the Medical Superintendent, Valkenburgh Hospital for the Insane, Capetown.

*Military and Naval.*

(261) 1. Can you inform me to whom to apply for particulars concerning Army and Navy Nursing Services ? 2. Also if appointments are given in rotation to accepted candidates, or obtained by influence.—*Humbers.*

Apply to the Under-Secretary of State, War Office, Pall Mall S.W., for particulars as to Army Nursing Service; and to the Director-General, Medical Department of the Navy, Admiralty, Craven House, Northumberland Avenue, W.C., for those relating to the Navy Staff of Nursing Sisters. 2. By rotation.

*Epileptic.*

(262) Will you kindly tell me of a home, free or nearly so, for an epileptic girl of thirteen ? She is a labourer's daughter.—*J. C. E.*

The National Society for the Employment of Epileptics, Chalfont St. Peter's, Bucks (office, 12 Buckingham Street, Strand, W.C.), has a home for girls.

*Egypt.*

(263) I wish to join an association in Alexandria or Cairo : will you tell me the best way of gaining information ; also the best time to go to either of these places ?—*Dutch.*

You might write to Miss Cutler, Victoria Nursing Home, Cairo ; and to Lady Superintendent of the Nurse's Home at Alexandria. The beginning of winter is the best time to go.

*India.*

(264) Could you kindly tell me if there are civil appointments in India for English nurses ?—*Mary O'N.*

The Up-Country Nursing Association for Europeans in India, Hon. Sec. Major-Gen. J. Bonus, The Cedars, Strawberry Hill, and the Ceylon Nurses' Association both employ English nurses. The Colonial Nursing Association, Imperial Institute, S.W., might also help you.

*Supplementary.*

(265) Can you tell me where I could supplement my midwifery training (Q.C.H. and L.O.S.) with general nursing for one year ? Salary expected.—*M. H.*

You can receive no salary unless you give two years' time. See the "Nursing Profession : How and Where to Train," for list of hospitals devoted to women's complaints.

*District Work.*

(266) Is it usual for district nursing associations to provide poor patients with bed linen and apparel, and, if so, what articles are provided ?—*Novice.*

It is generally considered advisable that nursing associations should only provide nurses. Further aid when necessary being supplied by distinct charitable agencies.

*Visiting Cases.*

(267) I am very anxious to get on as visiting nurse. I enclose one of my professional cards, and should be glad to know if it is all right. If it is not, would you kindly suggest any alteration.—*Nurse R.*

Your cards and forms are very nicely got up, and should answer the purpose admirably.

*Massage.*

(268) Could you kindly tell me of a good book on massage. Also the price of it ?—*E. D. P.*

"The Art of Massage," by Mrs. Creighton Hale, price 6s.

*Home for Aged.*

(269) Can you tell me of a home for a woman of sixty, who is suffering from creeping paralysis. She requires nursing, and can afford to pay 10s. weekly ?—*G. I. M.*

She seems a suitable case for the Hospital for Epilepsy and Paralysis, 32 Portland Terrace, Regent's Park, N.W.

*Male Nurse.*

(270) I am twenty-two years of age, and have always been attracted towards nursing. Would you advise me to train as nurse, and, if so, to what hospital can I apply for training ? What are the prospects of a trained male nurse ?—*Advice.*

The prospects are good. The National Hospital for the Paralysed and Epileptic, Queen's Square, Bloomsbury, W.C., offers a two years' course of training with salary to male nurses.

*Maternity.*

(271) Could you kindly inform me where a young lady could get a berth as probationer in accouchement cases without paying premium ?—*J. C.*

None of the lying-in hospitals charge a premium ; but none give free training. See advertisements in MIRROR for address of hospitals ; or consult the "Nursing Profession : How and Where to Train" for full particulars of terms of training.

*Sufficient Training.*

(272) Will a one year's certificate in general nursing from a good training school, with a two months' training in maternity work, qualify me for private cases ? Would a doctor consider the training sufficient ?—*K. A.*

Certainly it is sufficient, but a three years' certificate is better.

*L.O.S.*

(273) T. B. S. would like to know the date of the next L.O.S. examination. Does the society supply preparatory papers to the candidates ?

All information can be obtained from the Secretary, L.O.S., 20 Hanover Square, W. The next examination is in April.

*Surgical Instruments.*

(274) Can you tell me where to obtain a good book, which gives lists of instruments for different operations ?—*Anxious.*

Any of the firms of instrument makers advertising in the MIRROR would send you an illustrated catalogue if you sent your professional card and asked for one.

*Night Nurse.*

(275) Is it usual for nurses on night duty to attend the ordinary day lectures to probationers, or is a special course given to suit their hours of duty ?—*G. P.*

It is a matter entirely for the matron to arrange.

*Abroad.*

(276) 1. Will you kindly tell me of any agency in town by which nurses may obtain work abroad ? 2. What is the name of the paper which confines itself to asylum appointments ?—*Sister Mary.*

Appointments are secured by all the good associations for members desirous of going abroad. The Colonial Nursing Association, Imperial Institute, S.W., works entirely in that direction. 2. The "Asylum News," The County Asylum, Lancaster.

*Military Hospital.*

(277) Would you kindly tell me how long I should have to train, and if it is necessary to pass a dentist, before going to a military hospital ?—*N. M.*

A three years' certificate from a good civil hospital is an essential qualification for the Army Nursing Service. See the "Nursing Profession : How and Where to Train," for full particulars of the Government services. The regulations are too long to quote.

*So-called Registration.*

(278) Kindly tell me where nurses apply for registration. Is there any fee ? Do nurses receive a badge or certificate on registration ?—*Margaret.*

We presume you refer to membership of the Royal British Nurses' Association. Yes, the members receive a certificate and wear a badge. You will receive full information upon applying to the Secretary, 10 Orchard Street, W.

*Medical Etiquette.*

(279) Is it usual for the doctors of an infirmary or hospital to continue to attend and prescribe for their patients after they have been admitted to a private convalescent home, situated two miles from the institution, and under the care of its appointed medical attendant ?—*Nurse D.*

Patients in private homes have the privilege of selecting their own medical men.

*Advertisements.*

(280) In what paper are the vacancies of convalescent homes for matrons advertised ? I have searched the MIRROR for months, and have only seen one where a salary is given.—*C. S.*

Vacancies are often filled up privately, or from the permanent staff. Vacant posts are sometimes advertised in local papers ; but the MIRROR is recognised as the best medium for this class of appointment.

*Gynæcological.*

(281) Can you kindly tell me where a trained nurse can get a three or six months' course of gynæcological training ?—*Nurse E. W.*

The Hospital for Women, Soho Square, W., and the Chelsea Hospital for Women, Fulham Road, S.W. Both offer short courses of training to paying probationers. Possibly a trained nurse might obtain more favourable terms.

*Untrained Maternity Nurses.*

(282) Can a nursing association, or the trained midwife employed by it, prevent an unqualified woman taking maternity cases and charging full fees ?—*Justice.*

No one can prevent a woman nursing maternity cases and charging what fees she can get.

*Diphtheria.*

(283) Will you kindly tell me how infection is conveyed in diphtheria. Should the isolation of the attendant be as strictly observed as in scarlet fever ?—*Muddled.*

The infection of diphtheria spreads from patient to patient, and it is probably conveyed by the transference of infective particles from case to case ; hence the importance of the attendants using proper antiseptic precautions in nursing.

*Examinations.*

(284) Would the certificates of a hospital where no examinations are held such as the "Middlesex" be equivalent to one from a hospital where it is essential to pass such an examination ?—*Intending Probationer.*

It entirely depends upon the reputation of the institution. The Middlesex Hospital nurses suffer no loss in their profession because there is no formal examination at the end of their training.

*Assistant Nurse.*

(285) Can you tell me of any hospital where I could be taken on as assistant nurse ? I have been a children's nurse, and I am strong and healthy though obliged to wear glasses.—*Laura M.*

Do you mean assistant nurse, or probationer ? See the advertisements in the MIRROR, and apply for any that seem suitable. To become a properly trained nurse you should enter an infirmary as probationer for a three years' training.

### Standard Books of Reference.

**"The Nursing Profession : How and Where to Train." 2s. net ; post free 2s. 4d.**

**"The Nurses' Dictionary of Medical Terms." 2s.**

**"Burdett's Series of Nursing Text-Books." 1s. each.**

**"A Handbook for Nurses." (Illustrated.) 5s.**

**"Nursing : Its Theory and Practice." New Edition. 3s. 6d.**

**"Helps in Sickness and to Health." Fifteenth Thousand.**

**"The Physiological Feeding of Infants." 1s.**

**"The Physiological Nursery Chart." 1s. ; post free, 1s. 3d.**

**"Hospital Expenditure : The Commissariat." 2s. 6d.**

**All these are published by THE SCIENTIFIC PRESS, Ltd., and may be obtained through any bookseller or direct from the publishers, 28 and 29 Southampton Street, London, W.C.**

